# Therapeutic potential of TNFR2 agonists: a mechanistic perspective

**DOI:** 10.3389/fimmu.2023.1209188

**Published:** 2023-08-17

**Authors:** Yibo Chen, Mengmeng Jiang, Xin Chen

**Affiliations:** ^1^ State Key Laboratory of Quality Research in Chinese Medicine, Institute of Chinese Medical Sciences, University of Macau, Macau, Macau SAR, China; ^2^ Ministry of Education (MoE) Frontiers Science Center for Precision Oncology, University of Macau, Macau, Macau SAR, China; ^3^ Department of Pharmaceutical Sciences, Faculty of Health Sciences, University of Macau, Macau, Macau SAR, China; ^4^ Guangdong-Hong Kong-Macau Joint Lab on Chinese Medicine and Immune Disease Research, Macau, Macau SAR, China

**Keywords:** Treg - regulatory T cell, TNFR2 agonism, TNFR2, tumor, autoimmune diseases

## Abstract

TNFR2 agonists have been investigated as potential therapies for inflammatory diseases due to their ability to activate and expand immunosuppressive CD4+Foxp3+ Treg cells and myeloid-derived suppressor cells (MDSCs). Despite TNFR2 being predominantly expressed in Treg cells at high levels, activated effector T cells also exhibit a certain degree of TNFR2 expression. Consequently, the role of TNFR2 signaling in coordinating immune or inflammatory responses under different pathological conditions is complex. In this review article, we analyze possible factors that may determine the therapeutic outcomes of TNFR2 agonism, including the levels of TNFR2 expression on different cell types, the biological properties of TNFR2 agonists, and disease status. Based on recent progress in the understanding of TNFR2 biology and the study of TNFR2 agonistic agents, we discuss the future direction of developing TNFR2 agonists as a therapeutic agents.

## Introduction

Tumor necrosis factor (TNF) is one of the most potent pro-inflammatory cytokines that cause cell death and promote inflammatory responses, and high levels of TNF are attributable to the pathogenesis of autoimmune disease (1, 2). Anti-TNF therapeutics have been used as a first-line biological treatment of a variety of inflammatory diseases, including rheumatoid arthritis (RA), psoriasis, and inflammatory bowel disease (IBD) (3–5). However, paradoxically autoimmune inflammation frequently occurred in a subset of patients who received anti-TNF treatment. For example, anti-TNF therapy can increase the incidence of multiple sclerosis (6). These observations brought intense interest in elucidating the cause and mechanism. TNF receptor type I (TNFR1) and TNFR2 are two different receptors that mediated the biological function of TNF (7). A recent report showed that polymorphisms in TNFR2 frequently occurred in patients with IBD or RA (8, 9), which suggests that TNFR2 signaling plays an essential role in preventing these diseases.

TNFR2 is preferentially expressed by immunosuppressive cells, including Tregs, MDSCs, and some endothelial progenitor cells (EPCs) (10). Now, there is compelling evidence showing that TNF-TNFR2 signaling plays an important role in curbing pro-inflammatory responses and promoting tissue regeneration. It was reported that TNFR2 deficiency aggravated autoimmune inflammatory responses in collagen-induced arthritis (CIA) (11), experimental autoimmune encephalomyelitis (EAE) (12), graft-versus-host diseases (GVHD) (13), and psoriasis (14). Moreover, several studies have shown that TNFR2 agonists protect mice from autoimmune inflammatory diseases and degenerative diseases (15–17). Thus, TNFR2 agonists have been proposed as a novel strategy for the treatment of autoimmune diseases, by mainly activating and expanding TNFR2-expressing Tregs, and MDSCs (18–20). However, some studies indicate that TNFR2 plays an important role in the activation of conventional T cells (Tcon cells) and CD8 T cells (21, 22). Furthermore, antibodies that can trigger the TNFR2 signal in vitro were shown to promote antitumor immune responses by activating CD8 T cells, Tcon cells, or NK cells (23). And consequently, TNFR2 agonists have been developed for the treatment of human cancers (19). Thus, the role of TNFR2 signal in orchestrating the inflammatory responses in autoimmune diseases or immune responses in cancers is complicated, and the mechanism may be involved in the activation of Tregs, MDSCs, CD8^+^ T cells and ADCC, or inversely, depletion of Tregs in tumor environment (summarized in **Table 1**). The contradictory pro-inflammatory and anti-inflammatory properties of TNFR2 signaling should be further clarified in future investigations. This contradictory pro-inflammatory and anti-inflammatory property of TNFR2 signaling should be further clarified in future investigation

**Table 1:** The application of TNFR2 agonist in autoimmunity and cancer.

Category	Class	Agent	In vitro activity	In vivo activities
Autoimmunity				
	**TNFR2 agonistic antibody**	“TNFR2 agonist”	a) Promote the expansion, immunosuppressive function, and phenotypic stability of human Tregs (24).	N/A
		“TNFR2 agonistic antibody**”**	a) Promote Treg expansion and immunosuppressive function (25) b) Promote fatty acid oxidation in Tregs (25)	N/A
		MR2-1(Isotype: mouse IgG1)	a) Promote the expansion and immunosuppressive function of Tregs (26–28). b) Promote CXCL13 expression on T follicular regulatory cells (26). c) Promote EZH2 expression in Tregs (29). d) Promote glycolysis in Tregs (23, 27). e) Promote cell death of autoreactive CD8 T cell death (30)	N/A
		TY010	a) Promote M2 polarization (12). b) Promote IFN-γ expression in NK cells (31).	N/A
	**Transmembrane mimetics**	STAR2	a) Promote the expansion, immunosuppressive function of Treg (15, 17, 32–34). b) Protects oligodendrocyte progenitor cells and nerouns from oxidative stress-induced cell death (35, 36).	Protects mice from collagen-induced arthritis (15), GvHD (33), , BCG-induced chronic inflammation (34)
		New STAR2 (STAR2 conjugated with IgG)	a) Promote Treg expansion and immunosuppressive function (17, 32, 37) b) Enhances Microglial Phagocytosis (17, 32)	Protects mice from Alzheimer’s disease (17) and GvHD (37)
		EHD2-scTNF_R2_	a) Promote the expansion, immunosuppressive function of Treg (38–40). b) Activating PI3K-PKB/Akt and NF-κB signaling (40–42)	Protects mice from neuropathic pain (39), collagen-induced arthritis (21), traumatic contusive injury (43), Experimental autoimmune encephalomyelitis (41), and Alzheimer’s disease (42).
		P53-sc-mTNF_R2_ and GCN4-sc-mTNF_R2_	a) Promote the expansion of Treg (44).	N/A
	**TNF mutants**	TNF07	a) Promote the expansion and immunosuppressive function of Treg (45, 46).	Protects mice from DNFB-sensitized contact hypersensitivity (46).
	**Endogenous proteins**	Membrane lymphotoxin-α2β	a) transmembrane LTα2β robustly activates human TNFR2 signaling (47).	N/A
		Progranulin (PGRN) or its derivatives.	Promotes TNF-induced Treg proliferation (48). Promotes M2 polarizaiton (49). Promotes the IL-10 expression (50, 51).	Protects mice from osteoarthritis (52, 53).
Cancer				
	TNFR2 antibody trigger TNFR2 activation in vitro	TNFR2 agonist (Y9)	Promote the activation of CD8 T cells and NK cells (54).	Inhibit the tumor growth (Require FcγR activity) (54).
		MM401	Provides T cell co-stimulation (55).	Inhibit tumor growth and deplete Treg with ADCC (56) (Require FcγR activity) (55, 56)
		BI1910	Promote CD8 T cell function and infiltration (57). Regulating the myeloid contents in tumor (57).	Inhibit tumor growth with or without IgG conjugation (57)
		HFB200301	Activates T cells, NK cells and Tregs in vitro (58).	Inhibit tumor growth without affecting Treg numbers (independent of FcγR activity) (58)
		IAT0981-231	stimulated CD8^+^ T cell activation, proliferation and cytokine secretion (59)	Inhibit tumor growth (59)

This review will focus on the discussion of the current understanding of the effects of TNFR2 agonists on inflammatory responses and anti-tumor immune responses. The development of TNFR2 agonists is introduced and the effect of these agents on the activation of different subsets of immune cells, and factors that may determine the therapeutic outcome of TNFR2 agonists in the treatment of cancer or autoimmune diseases, are reviewed and analyzed.

## Overview of TNFR2 agonists

### TNF mutants

Selective mutation of residues in TNF protein significantly altered its affinity to TNF receptors. The TNF mutant (D143N-A145R) is a TNFR2-selective agonist developed for decades. TNF mutant (D143N-A145R) only binds to TNFR2 but not TNFR1 (60). However, such a TNF mutant presents a 5~30 fold lower affinity to TNFR2 in comparison with wild-type (WT) TNF (61, 62) In recent years, several new TNF mutants that selectively bind and activate TNFR2 were developed by the phage display technique (63). The SPR analysis showed that these TNF mutants bind to TNFR2 with lower affinity but had a higher association/dissociation rate in contrast with WT TNF (63), indicating TNF mutants can form a stable complex with TNFR2Moreover, an *in-vivo* study showed that the TNF mutants fused with IgG could trigger the activation of TNFR2 signaling and induce Treg proliferation in a TNFR2-dependent manner (46). These results suggested that TNFR2-selective TNF mutants exhibit different binding modes for unique biological functions.

### Transmembrane TNF mimetics

**Figure 1:**
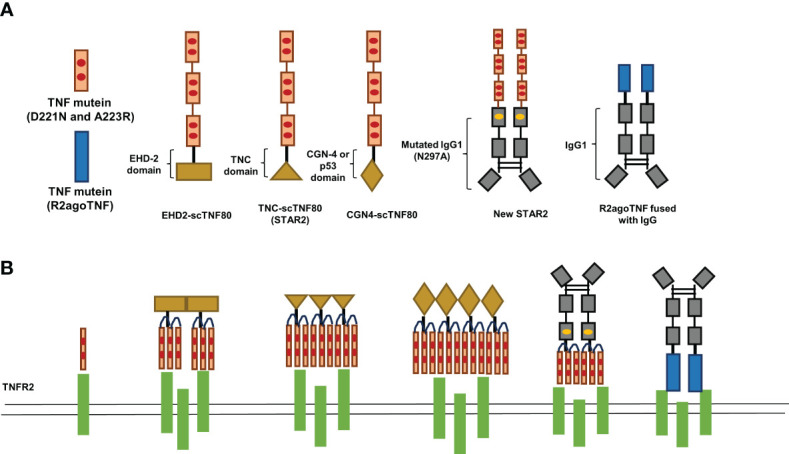
A schematic of TNF mutants and transmembrane TNF mimetics.

Compared with TNFR1, TNFR2 can only be fully activated by transmembrane TNF (64). The monomer transmembrane TNF always forms homotrimers on the cell membrane as a consequence of self-assembly before binding with TNFR2 (65, 66). Thus, one of the strategies to enhance the affinity of TNFR2-selective TNF mutants is to construct oligomerized TNFR2-selective TNF mutants (**Figure 1**). STAR2, a TNFR2 agonist composed of murine TNF mutants (D221N and A223R) and trimerization domain from chicken tenascin C, displays significantly higher affinity to TNFR2 than single chain TNF mutein and can induce TNFR2 activation more effectively (33). Moreover, STAR2 treatment significantly promoted Treg expansion in the mouse GVHD model (33). Based on this idea, Fisher et al. generated several oligomerized TNFR2-selective TNF mutants by using different oligomerization domains. The results showed that dodecavalent ligands by engineering oligomerization domain from GCN4 and TNFR2-selective TNF mutants (GCN4-sc-mTNF_R2_) displayed superior bioactivity and affinity than other oligomerized TNFR2-selective TNF mutants in vitro (44). Furthermore, GCN4-sc-mTNF_R2_ could be less immunogenic because the structure of GCN4-sc-mTNF_R2_ more resembles human protein structure (44). Although oligomerized TNFR2-selective TNF mutants represent a more effective strategy to evoke TNFR2 activation, the risk of immunogenicity cannot be neglected as the sequence of oligomerized TNFR2-selective TNF mutants cannot be found in nature. Thus, the immunogenicity of oligomerized TNFR2-selective TNF mutants should be carefully evaluated.

### Progranulin (Endogenous proteins)

Progranulin (PGRN) is a secreted factor that regulates biological processes including inflammation, wound healing, and tissue repair (67). The initial results showed that progranulin directly interacts with and antagonizes both TNFR1 and TNFR2 (68), but subsequently, studies indicated that progranulin triggers the activation, instead of blocking TNF-TNFR2 signaling (69). Moreover, progranulin exhibits a relatively high affinity to TNFR2 (68). It has been shown that progranulin or its derivatives, Asttrin, alleviated the inflammatory responses in a TNFR2-dependent manner (50, 52, 70). However, there is contradictory evidence that progranulin may not interact with TNFRs, as progranulin failed to block the TNFR1-induced cell death (71). Furthermore, different concentrations of progranulin (2~200 ng/ml) plus IL-2 did not promote Treg proliferation, indicating that progranulin may not directly agonize TNFR2 (48). Thus, further investigation is needed to clarify if progranulin indeed promotes the activation of TNF-TNFR2 signaling.

### TNFR2 agonistic antibodies

Recently, several TNFR2 antibodies with the capacity to activate TNFR2 *in vitro* have been developed for the treatment of autoimmune diseases (25) or cancers (57, 59, 72, 73). These TNFR2-stimulating antibodies were reportedly to possess either immunostimulatory or immunosuppressive *in vivo*, as its complicated nature, presumably based on different mechanism. For example, Y9, a close of agonistic anti-TNFR2 antibody, was found to be a competitive activator of TNFR2 and bind to CRD2 and CRD3 in TNFR2 (54). Other TNFR2-stimulating antibodies appeared to bind to CRD1 and CRD2 and did not compete with TNF for binding with TNFR2 (25, 57, 58). Whether the antibodies compete to bind to TNFR2 could also be important for their in vivo effect, as competitive activators sparing more TNF in local tissue, which may enhance the TNF-TNFR1 signaling. In contrast, the non-competitive TNFR2-stimulating antibodies did not affect TNF-TNFR2 interaction but may synergize with TNF in activating TNFR2 (74). Therefore, it is important to determine whether the non-competitive activator could elicit different responses with competitive activators.

Fragment crystallizable region (Fc) is another factor that profoundly affects the function of TNFR2-stimulating antibodies. For example, TNFR2 agonists may require FcγR activity for more potent agonistic function, as it confers TNFR2 agonist the transmembrane TNF-like activity (75). This was evidenced by the observation that TNFR2 agonistic antibody (BI-1910) with poor FcγR-binding activity (N297A, IgG1 mutated) exhibited a decreased antitumor effect as compared to IgG1 or IgG2a conjugated BI-1910 (57). Therefore, the engagement of FcγR could be an important factor that determine the therapeutic effect of some TNFR2 agonistic antibodies (76). Moreover, Fc conjugation can induce the antibody-dependent cellular cytotoxicity (ADCC) or antibody-dependent cellular phagocytosis (ADCP). For example, the antitumor effect of Y9 was reportedly dependent on the ADCC and ADCP, as its antitumor effect was diminished in Fcgr2b^−/−^ and Fcer1g^−/−^ mice (54). In this case, Y9 may deplete TNFR2-expressing cells rather than a TNFR2 agonist. Nevertheless, a recent study shows that a TNFR2 agonistic antibody had a more potent function in the presence of antibody that can block crosslinking activities (25), suggesting that neither ADCC nor Fc-mediated crosslink activities were required for the activity of this TNFR2 agonistic antibody.

## TNFR2 agonist-induced activation of immune suppressive cells

### CD4^+^Foxp3^+^ regulatory T cells

There is compelling evidence that TNFR2 plays a pivotal role in Treg activation, function, proliferation, and phenotypic stability (77, 78). Several TNFR2 agonists have been reported to alleviate inflammatory responses by promoting Treg function and expansion (15, 33, 34, 39, 40). TNFR2 agonism also represents an efficient approach to expand the Tregs from low-purity human Tregs for adoptive Treg transfer therapy. Several studies showed that TNFR2 agonistic antibody plus the standard Treg expansion protocol (in the presence of CD3/CD28, IL-2 with or without rapamycin) resulted in the expansion of homogenous stable Tregs with potent immunosuppressive function (24, 25, 33). Moreover, TNFR2 agonistic antibody treated-Treg has lower expression of CD127, IL-17A, and IFN-γ, indicating TNFR2 agonistic antibody help maintain the phenotypic stability of expanded Tregs (79). It is of great interest to examine whether TNFR2-agonist-expanded Tregs are more effective for adoptive Treg transfer therapy.

### Myeloid-derived suppressor cells

MDSCs, a subset derives from pathologically activated neutrophils or monocytes, have potent immunosuppressive activity. MDSCs are considered a potential target for the treatment of cancer and autoimmune diseases (80). It was reported that TNF-TNFR2 signaling is important for the recruitment, immunosuppressive function, and survival of MDSCs (81–83). Thus, MDSCs is also a potential target of TNFR2 agonist. However, recent studies showed that the effect of TNFR2 agonists is mainly mediated by CD4 T cells, albeit with a minor effect on MDSCs. Lamontain and colleagues reported that TNFR2 agonist (TNCscTNF80) treatment promoted the expansion of MDSCs in bone marrow, but not in spleen and lymph nodes in the mouse CIA model, indicating the anti-inflammatory effect of TNCscTNF is not dependent on MDSCs (15). This result was further supported by the data from CD4^cre^TNFR2^fl/fl^ and LysM^cre^TNFR2^fl/fl^ mice. TNFR2 agonist (TNCscTNF80) suppresses T cell proliferation in LysM^cre^TNFR2^fl/fl^ mice but not in TNFR2^-/-^ mice and CD4^cre^TNFR2^fl/fl^ (34), indicating the anti-inflammatory effect of TNFR2 agonist were mainly dependent on the TNFR2 expression by CD4 T cells. Further evidence is needed to support the claim that TNFR2 agonists can boost MDSC’s activity to suppress inflammatory responses.

### Monocytes/macrophages

Monocytes and macrophages express both TNFR1 and TNFR2. These two receptors play complicated roles in the regulation of the viability, function, and recruitment of monocytes/macrophages (84). Moreover, tissue-resident macrophages may also have different responses to TNFR2 activation, as compared with circulating monocytes/macrophages (85). Thus, the effect of TNFR2 agonism on macrophages could be tissue specific. It has been shown that administration of TNFR2 agonist (EHD2-sc-mTNFR2) increased the expression of M2 markers in macrophages and macroglia, and reduced the expression of M1 markers, but without activation of macrophage in mouse central nervous system (16, 39). However, these effects of TNFR2 agonists could be the indirect result of the activated Tregs which may suppress macrophage activity (86). A recent study showed that TNFR2 agonist (TY010) promoted M2 polarization of bone marrow-derived macrophage in TNFR2 dependent manner, indicating TNFR2 agonist may directly activate TNFR2 on macrophage and induced an immunosuppressive phenotype (49). Moreover, TNFR2 agonist (NewStar2, TNCscTNF80 fused with mutated human IgG) has been shown to enhance the phagocytosis of microglia and promote the clearance of Aβ plaques, which contributes to the alleviation of Alzheimer’s diseases in mouse (17). These results indicate that macrophages/monocytes are the targets of TNFR2 agonists in the treatment of inflammatory diseases.

## TNFR2 agonist-induced activation of effector immune cells

### Conventional T cells

TNFR2 has been shown to promote the activation, function, differentiation, and proliferation of Tcon cells (22, 87, 88). TNFR2^+^ Tcon cells are more resistant to Treg-mediated immunosuppression (87). However, TNFR2 was expressed much lower by Tcon cells than by Tregs in the resting state (87). Thus, TNFR2 agonists may not effectively activate TNFR2 signaling in unstimulated Tcon cells (89). Previous studies showed that the treatment with TNFR2 agonists (TNF mutants or transmembrane mimetics) inhibited Tcon cell proliferation by promoting Treg expansion (32). However, TNFR2 expression can also be upregulated by TCR stimulation (90) or pro-inflammatory cytokines (91), suggesting the activated Tcon cells could respond to TNFR2 agonists. A recent study showed that stimulation of anti-CD3 and a TNFR2 agonistic antibody (MR2-1) induces a similar alteration of transcriptome profile, albeit the alteration of the Treg cell transcriptomic profile is more obvious (23). This effect of TNFR2 agonism has shown to be pathological-relevant, as TNFR2 is expressed by tumor-infiltrating Tcon cells (89, 91–93) and proinflammatory subsets of CD4 Tcon cells (94, 95), suggesting that TNFR2 agonists could induce the activation of TNFR2 signal in Tcon cells in tumor and inflammatory diseases. In the mouse tumor model, it was reported that TNFR2 agonistic antibodies induced the expansion of CD4^+^ Tcon cells without affecting the Treg number in vivo (58). Therefore, Tcon cells are also a potential target of TNFR2 agonists albeit with relatively lower TNFR2 expression.

### CD8 T cell

As one of the co-costimulatory receptors, TNFR2 promotes the activation, function, proliferation, differentiation, and recruitment of CD8 T cells (96–98). However, TNFR2 signaling can also play a dual role in the modulation of the activation of CD8 T cells. For example, genetic ablation of TNFR2 impairs the production of effector cytokine while can also result in a more persistent activation of CD8 cells in mouse tumor and infection models (99, 100). CD8 T cells at different stages of activation may likely respond to TNFR2 activation differently. This notion is supported by the observation that the activation of TNFR2 promotes the differentiation of naïve CD8 T cells (96), while TNFR2 stimulation also selectively induced the activation-induced cell death (AICD) of the autoreactive CD8 T cells without significantly affecting the other T cell subsets (30). The different responses could be attributable to the alteration of downstream signaling. TNFR2 expression is crucial for the activation of NF-κB signaling in CD8 T cells when stimulated with anti-CD3/CD28. While a persistent activation of TNFR2 can inhibit NF-κB signaling through depleting TRAF2, an important signal component in mediating NF-κB activation (39), thereby sensitizing CD8 T cells to TNF-induced cell death (101).

Although treatment of TNFR2 agonistic antibody can stimulate the activation of tumor-infiltrating CD8 T cells (57), the time frame of TNFR2 agonist treatment in a preclinical mouse tumor model is relatively short. Such studies may not be able to reflect the effect of long-term activation of TNFR2, including activation-induced cell death (AICD) or exhaustion of CD8 T cells (100, 102). More recently, we reported that TNFR2 expression is associated with the exhaustive phenotype of CD8 T cells in human cancers (103). Thus, the role of TNFR2 in tumor-infiltrating CD8 CTLs is complex and needs further investigation. A more thorough understanding of the molecular basis underlying the effect of TNFR2 signal in CD8 CTLs is crucial to device TNFR2 agonists in tumor immunotherapy.

### Natural killer cells

TNFR2 has been reported to be expressed by both human and mouse NK cells, albeit the expression pattern could be different (89). It has been shown that genetic ablation of TNFR2 has been shown to significantly decrease the expression of IFN-γ in α-galactosylceramide (α-GalCer)-treated mouse, indicating that TNFR2 signaling is also important for the activation and function of NK cells (31). TNF or TNFR2 agonist (TY010) in concert with IL-12 elevated the expression of IFN-γ in human and mouse NK cells in vitro (31, 104). The antitumor effect of TNFR2-targetingantibody (Y9) can be impaired by the depletion of NK cells, suggesting that TNFR2 agonists may also target the NK cells to elicit antitumor immune responses (54). Now several TNFR2-stimulating antibodies in clinical development have been reported to enhance NK cell activation (58, 73). However, the mechanism that how TNFR2 agonism affects tumor-infiltrating NK cells remains to be investigated.

## Factors may determine the therapeutic outcome of TNFR2 agonists in the treatment of cancer or autoimmune diseases

### TNFR2 expression: pro-inflammatory vs anti-inflammatory cell subsets

TNFR2 signaling can result in both anti-inflammatory or pro-inflammatory effects, depending on the cell type of TNFR2 expression and the functional status of the cells. High levels of TNFR2 are constitutively expressed by Tregs, and the activation of TNFR2 signaling in Tregs or autoreactive CD8 T cells (and other immunosuppressive cells) can cause immune suppression or elicit an anti-inflammatory effect (30, 78). However, elevated TNFR2 expression can be shown in pathogenic T cell subsets in patients with Crohn’s disease (94) and rheumatoid arthritis (95), and blockade of TNF promoted cell death of pathogenic T cells (94, 95). These results indicated that TNFR2 agonism may also promote pathogenic T-cell responses. The pathogenic T cells with elevated TNFR2 expression can be more resistant to Treg-mediated immune suppression (105). Moreover, activation of TNFR2 also promotes the inflammatory responses of innate immune cells and non-immune cells (106–108). The dual role or bi-phasic effect of TNFR2 signaling is exempted by a study that shows that, in TNFR1 deficient mice, infusion of murine TNF at the initial phase of collagen-induced arthritis increased the disease severity, while the same treatment markedly alleviated the inflammation in the progression phase (109). This study demonstrated the bi-phasic effects of TNF-TNFR2 signaling in an inflammatory response.

Both humanized TNFR2 antagonists and agonists have been developed for the treatment of tumors (19, 110), based on the notion that antagonistic antibodies may eliminate the immunosuppressive Tregs, while antibodies that trigger TNFR2 signaling may activate CD8 CTLs and NK cells (54, 56–58, 72). Despite the assumptions are opposite, the results of the studies appear to support that TNFR2 antagonistic antibodies inhibit the tumor infiltration of Tregs and consequently enhance the antitumor immune responses (111–119), while antibodies that trigger the TNFR2 signal in vitro also elicited antitumor immune responses (54, 56–58, 72). It was shown that TNFR2 activation promotes the differentiation and the production of effector cytokine by CD8 T cells (96, 120). Thus, as a costimulatory molecule, TNFR2 is likely to promote the initiation of antitumor T cell immune responses. In line with this notion, preclinical studies showed that TNFR2 agonistic antibodies with diminished activity to induce ADCC can enhance the antitumor immune responses by activating CD8 T cells and NK cells. However, it should be noted that the preclinical studies about TNFR2 agonistic antibodies were based on the transplanted tumor models and the time of experiment settings is relatively short. Such studies may not be able to reflect the effect of long-term activation of TNFR2, including activation-induced cell death (AICD) or exhaustion of CD8 T cells (100, 102).

### Ligand-based agonists vs agonistic antibodies

Different types of TNFR2 agonists may own distinguished features and consequently elicit different effects on immune response in vivo. We summarized the antibodies that can trigger TNFR2 signaling from the published studies (**Table 1**). Interestingly, some of these TNFR2 antibodies can enhance the anti-tumor immune responses even though they have been shown to significantly promote Treg expansion in vitro. While the TNFR2-selective TNF mutant or transmembrane TNF mimetics elicited anti-inflammatory responses in vitro and vivo. The paradoxical effects of antibodies were also reported in other TNFRSF members. For example, one of the GITR antibody has been shown to promote Treg expansion in vitro (121). However, this GITR antibody has also been shown to enhance the infiltration of non-Foxp3 expressing T cells into tumor tissue and enhance the antitumor immune responses (122). These results suggest the complicated nature of TNFR2-antibodies with stimulating activities. By comparing with transmembrane TNF mimetics, TNFR2-antibodies with agonistic activities also bind with TNFR2 with high affinity. However, the function of TNFR2-stimulating antibodies is significantly affected by the Fc region as aforementioned. Moreover, although there is compelling evidence that TNFR2 agonists promote the expansion of Tregs, excessive or prolonged TNFR2 activation may elicit different responses of Tregs in some circumstances. For example, tumor-infiltrating Tregs are highly activated and express high levels of TNFR2. The activation of TNFR2 may induce the TRAF2 depletion in Tregs present in the tumor environment, thus sensitizing Treg to TNF-induced cell death (123, 124). Moreover, although optimal PI3K/Akt/mTOR signaling could be important for TNF-induced Treg activation and expansion (26, 28), a high dose of TNFR2 agonistic antibody or superclustering of TNFR2 may also induce hyperactivation of PI3K/Akt/mTOR signaling pathway, which can destabilize Foxp3 expression (125). The agonistic antibody-induced Foxp3 instability and Treg cell death have been reported in other TNFRSF members with a similar co-stimulatory capacity (126, 127). Therefore, this evidence may provide an alternative interpretation of the reported anti-tumor effect of TNFR2 agonistic antibodies.

### Tissue-specific responses to TNFR2 agonists

The TNFR2 agonist-regulated immune responses could be tissue-specific, as the tissue-resident immune cells and non-immune cells can express TNFR2. TNFR2 activation may induce anti-inflammatory responses or pro-inflammatory responses as well, depending on the cell types targeted. There is compelling evidence that TNFR2 signaling not only suppresses the inflammatory responses in the central nervous system (CNS) but also promotes tissue repair of the CNS system by activating TNFR2 signaling through several different cell types (35, 128, 129). Thus, TNFR2 agonism evokes immunosuppression and tissue repair in the CNS. However, TNFR2 also induced potent inflammatory responses in some organs. For example, activation of TNFR2 signaling in parenchymal cells was important for the development of hepatitis in mice (130). Blockade of TNFR2 has been shown to alleviate anti-PD-1-induced hepatic inflammation in mouse hepatic carcinoma, even infiltration of Tregs was also decreased (131). Moreover, TNFR2 signaling in some tumor cell types also contribute to tumor progression (132, 133), so the potentially undesirable effect of TNFR2 agonistic antibody should be considered. Thus, tissue-specific responses are also an important factor that needs to be considered in TNFR2 agonist therapy.

## Conclusion and future perspective

Numerous studies indicated the potential of TNFR2 agonism in the treatment of autoimmune inflammatory diseases and cancer, as TNFR2 has a dual role in modulating immune responses. TNFR2 plays a decisive role in maintaining Treg function and activity, which is important for the suppression of autoimmune inflammatory responses. On the other hand, TNFR2 activation in Tcon cells or CD8 T cells also elicits pro-inflammatory responses. However, the therapeutic outcome of TNFR2 agonism could be affected by the property of agonists, the disease condition, and tissue-specific responses. To minimize the unwanted effect elicited by TNFR2 agonism, one of the strategies is to develop therapeutics that specifically target certain cell types. For example, IL2-EHD2-sc-mTNFR2, a recombinant protein fused with TNFR2 agonist (EHD2-sc-mTNFR2) and IL-2, induced a more potent Treg expansion than IL2 plus EHD2-sc-mTNFR2 (38). Moreover, combining the immunosuppressants with TNFR2 agonists could be another strategy in the treatment of autoimmune inflammatory diseases, as Tregs are more resistant to immunosuppressant-mediated cell death (134). Although several TNFR2 agonistic antibodies were demonstrated in the clinical trial, how the TNFR2 agonistic antibody elicits a different immune response in vivo remains largely unknown. Identifying the factors that affect biological consequences induced by TNFR2 agonists may pave the way to the more effective treatment of cancer or autoimmune diseases.

## Data availability statement

The original contributions presented in the study are included in the article/supplementary material. Further inquiries can be directed to the corresponding author.

## Author contributions

YC and MJ drafted the manuscript. XC, the corresponding author, was involved in designing the frame of the manuscript and approving the final version to be published. All authors contributed to the article and approved the submitted version.
